# Comparative Urodynamic Study in Cadaver of Urethral Pressure Profilometry Between the Artificial Urinary Sphincter UroActive and the AMS800

**DOI:** 10.1111/aor.70032

**Published:** 2025-10-24

**Authors:** Aurélien Beaugerie, Elliot Tokarski, Anne Denormandie, Juliette Cotte, Stéphanie Tran, Emmanuel Chartier‐Kastler, Pierre Mozer

**Affiliations:** ^1^ Department of Urology Pitié‐Salpêtrière Academic Hospital (AP‐HP) Paris France; ^2^ Sorbonne University Paris France

**Keywords:** artificial urinary sphincter, stress urinary incontinence, urodynamics

## Abstract

**Introduction and Objective:**

The artificial urinary sphincter (AUS) is currently the gold standard treatment for stress urinary incontinence in men, and it's also a treatment option for women in Europe. UroActive is a new electronic device that offers remotely adjustable settings, including device pressure. This study aims to compare the range of Maximal Urethral Closure Pressures (MUCPs) covered by UroActive with those covered by the current AMS800 in male and female cadavers.

**Methods:**

Six cadavers (3 males and 3 females) were implanted with an occlusive cuff (OC) positioned around the bulbar urethra in men and around the bladder neck in women. A MUCP measurement was performed for each of the 3 different AMS800 Pressure‐Regulating Balloons (PRBs): 51–60, 61–70, and 71–80 cmH_2_O, that were successively connected to the OC. The AMS800 PRB was then replaced by the UroActive Control Unit (CU), and MUCP measurements were performed at set pressures from 10 to 150 cmH_2_O.

**Results:**

UroActive device remained fully functional (wireless communication, calibration) throughout the study period. UroActive CU achieved MUCPs values that encompassed the entire range observed with the 3 different AMS800 PRBs. A strong positive correlation between set device pressures sent to UroActive CU and MUCPs was noted in both males (*r* ^2^ = 0.984) and females (*r* ^2^ = 0.948).

**Conclusion:**

The findings suggest that UroActive provides a wide adjustable range of urethral closure pressures, potentially offering an alternative to AMS800 for managing stress urinary incontinence in both men and women. Further clinical studies are necessary to confirm its safety and effectiveness in patients.

## Graphical abstract

The Artificial Urinary Sphincter is currently the gold standard treatment for stress urinary incontinence in men, and it's also a treatment option for women in Europe. UroActive is a new electronic AUS that offers remotely adjustable settings, including device pressure, potentially offering an alternative to the current AMS800.

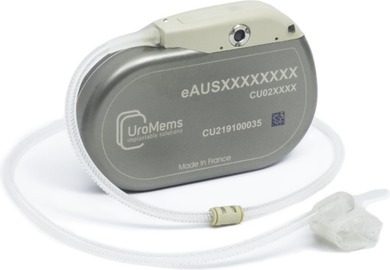

## 1 Introduction

Stress urinary incontinence is a frequent pathology in both men and women [1, 2] that can lead to a significant social handicap for patients [3]. The Artificial Urinary Sphincter (AUS) is currently the gold standard treatment for stress urinary incontinence in men [4, 5]. It is also a treatment option for women in Europe, particularly in the case of those with sub‐urethral slings failure [6].

The most widely implanted AUS in Europe and the United States is the AMS800 (Boston Scientific, USA). This is an implantable medical device composed of three parts, including a Pressure‐Regulating Balloon (PRB) which pressurizes the system at a constant pressure. The PRB is selected during the surgery and cannot be changed after implantation. There are 3 different PRBs available on the market, the 61–70 cmH_2_O being the most commonly used.

For many years, different AUS have been imagined and developed with the aim of improving the safety, efficiency, and usability of the current device [7, 8, 9]. The UroActive AUS (UroMems, France) is an implantable device that embeds electronic components and a radiofrequency antenna, enabling it to communicate wirelessly with an external programmer. This feature allows the physician to remote adjust device parameters, including device pressure, even after implantation surgery. UroActive is not approved yet for clinical use, but its biocompatibility and safety have already been tested on large animals [10].

The primary objective of this pre‐clinical study is to evaluate the range of maximal urethral closure pressures (MUCP) achievable by UroActive compared to those of the AMS800 in male and female cadavers. The secondary objective is to assess the correlation between the set pressure and MUCP with the UroActive device.

## 2 Methods

This study was conducted on fresh cadavers at the Surgical and Anatomical School of the Assistance Publique—Hôpitaux de Paris (FR), following approval by the local ethics committee.

First, an AMS800 OC was implanted using the usual technique: around the bulbar urethra via a perineal approach in males and around the bladder neck via a suprapubic laparotomy in females. OC size was selected using the cuff sizer provided in the AMS800 accessory kit. After being purged with isotonic saline solution, OC was placed around the bulbar urethra/bladder neck and its tubing was routed to the right part of the abdomen. Incisions were closed.

Then, the 3 different AMS800 PRB: 51–60, 61–70, 71–80 cmH_2_O were sequentially connected to the OC with a suture‐tie connector provided in the accessory kit. They were prepared and filled with 20 cc of isotonic saline solution. For each PRB, a Urethral Pressure Profilometry (UPP) was performed using the Goby urodynamic station (Laborie, USA) with a 10Fr 2‐way Bohler urethral catheter.

**FIGURE 1:**
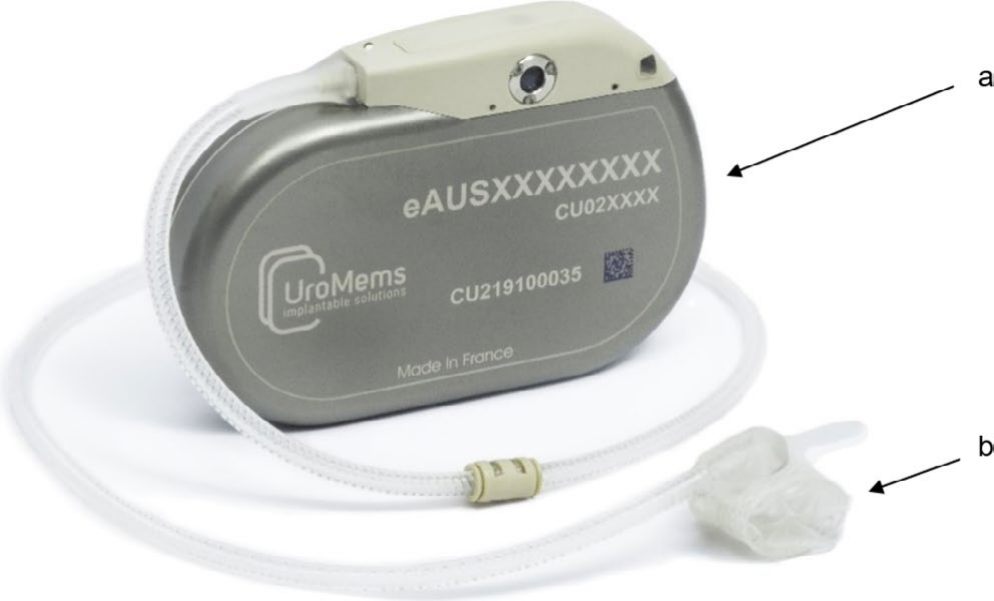
UroActive device (a, control unit; b, occlusive cuff). [Color figure can be viewed at wileyonlinelibrary.com]

After these 3 UPPs, AMS800 PRB was replaced by the UroActive Control Unit (CU). The CU is a titanium case (see attached Figure 1) that embeds a fluidic reservoir, an automatic pump, a battery, electronic components, and a radiofrequency antenna allowing it to communicate wirelessly.

The CU was previously filled with isotonic saline solution and then connected to the implanted OC with a suture‐tie connector. The CU was placed at the same level as the previous PRBs to minimize the influence of the water column above the OC. After device volume/pressure calibration, pressure was set in the UroActive device to close the cuff and UPP was performed. Multiple UPPs were carried out for CU set pressures ranging from 10 to 150 cmH_2_O, with steps of 10 cmH_2_O.

The endpoint used was the MUCP, defined as the highest pressure measured during UPP.

For the primary objective, the MUCPs achievable by UroActive were compared to those of the AMS800 in male and female cadavers.

For the secondary objective, a Pearson correlation analysis was conducted on male and female cadavers to assess the relationship between the device pressures (PRB specification for AMS800; set CU pressure for UroActive) and MUCPs. Statistical analyses were performed using the XLSTAT software, version 2023.1.1 (Addinsoft, New York, USA).

## 3 Results

Six cadavers were tested, including 3 males and 3 females.

AMS800 OCs, PRBs, and UroActive CU were implanted without any difficulty. OC sizes were 4, 4, and 4.5 cm for males, and 5, 5, and 5.5 cm for females.

For UroActive device, wireless communication between the CU and the clinician programmer was trouble‐free and device calibration was performed without any difficulty in all cadavers.

**FIGURE 2:**
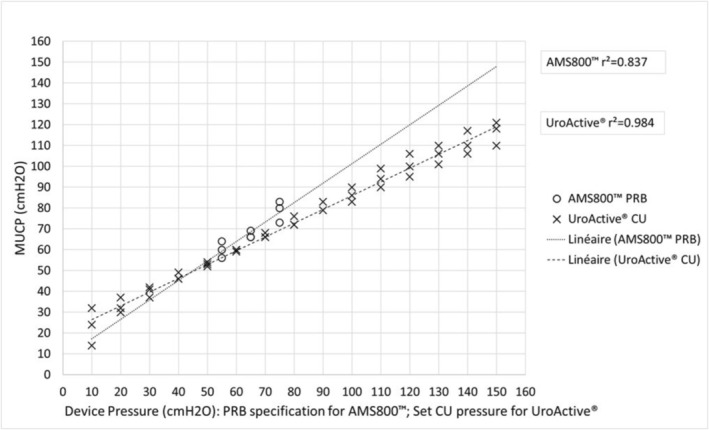
MUCP according to device pressure in male cadavers.

**FIGURE 3:**
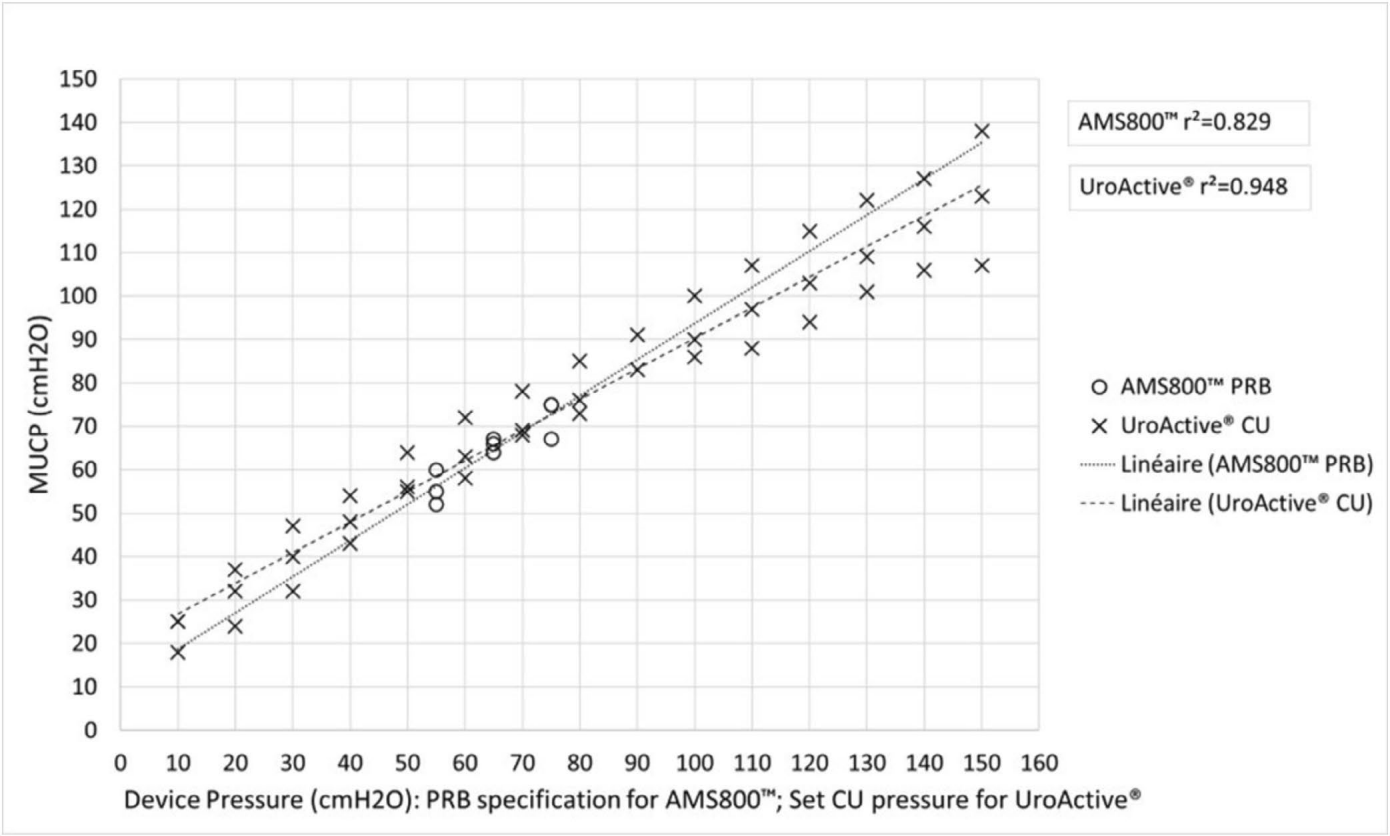
MUCP according to device pressure in female cadavers.

Results are represented in Figure 2 for males and Figure 3 for females, and detailed below.

### 3.1 Primary Objective

For male:

- With the AMS800 PRBs, MUCPs ranged from a minimum value of 56 cmH_2_O with the 51–60 cmH_2_O PRB to a maximum value of 83 cmH_2_O with the 71–80 cmH_2_O PRB.
- Using the UroActive CU, MUCPs ranged from a minimum of 14 cmH_2_O at a set pressure of 10 cmH_2_O to a maximum of 121 cmH_2_O at a set pressure of 150 cmH_2_O.

For female:

- With the AMS800 PRBs, MUCPs ranged from a minimum value of 52 cmH_2_O with the 51–60 cmH_2_O PRB to a maximum value of 75 cmH_2_O with the 71–80 cmH_2_O PRB.
- With the UroActive CU, MUCPs ranged from 18 cmH_2_O at a set pressure of 10 cmH_2_O to a maximum value of 138 cmH_2_O at a set pressure of 150 cmH_2_O.

These MUCP measures show that the UroActive control unit achieved MUCPs that encompass the full range of values obtained with the three AMS800 PRBs for both males and females.

### 3.2 Secondary Objective

For AMS800:

- The correlation coefficient between the PRB pressure‐specification and MUCPs is *r* ^2^ = 0.837 for males and *r* ^2^ = 0.829 for females.

For UroActive:

- The correlation coefficient between the set device pressure send to the UroActive CU and MUCPs is *r* ^2^ = 0.984 for males and *r* ^2^ = 0.948 for females. So a strong correlation was observed between UroActive set device pressures and MUCPs in both genders.

## 4 Discussion

### 4.1 Urodynamic Method

Urethral occlusion can be assessed using various urodynamic methods, including urethral profilometry and leak point pressures, such as Retrograde Leak Point Pressure (RLPP) and Antegrade Leak Point Pressure (ALPP). During the development of this study's design, we evaluated these methods on cadavers in unpublished pilot studies. The RLPP, measured using the Comiter technique [11], provided highly relevant and reproducible results in male cadavers [12, 13]. However, its use in females was limited by the short length of the urethra, which prevented sufficient catheter positioning away from the OC while maintaining an effective seal. We also tested ALPP measurements with bladder filling through transcutaneous or urethral catheters [14], but reliable and reproducible results could not be achieved. Consequently, we have chosen UPP as the urodynamic reference method in this study. This method has already been used in clinical studies to compare AUS in vivo [15, 16].

### 4.2 Study Design

The implantation site of the OC differs significantly between males and females, leading to varying tissue properties and thickness inside the cuff (urethra vs. bladder neck). Therefore, we have chosen to separate the results by gender. Moreover, for females, OC sizes appear to be smaller in our study than in current clinical practice. In fact, monocentric series report a median implanted cuff size of 7 cm in women, whether implanted by laparotomy [17] or laparoscopy [18]. This is probably due to the absence of vascularization and tissue atrophy in the cadaveric model. This finding is not expected to affect MUCP measurements.

In this study, only the AMS800 OC was used. Consequently, the evaluation of the UroActive sphincter was conducted with a hybrid system composed of the UroActive CU and the AMS800 OC. This approach was chosen to limit potential biases related to changes in OC positioning, allowing us to focus solely on comparing the device pressurization systems: BRPs for the AMS800 versus CU for UroActive.

We limited the study to a maximum device pressure of 150 cmH_2_O with UroActive, in line with its technical specifications, as we believe that applying higher pressures in clinical practice could carry a significant risk of erosion. To balance precision and practicality, we increased the pressure in increments of 10 cmH_2_O, though finer adjustments are technically possible.

### 4.3 UroActive Device

UroActive is composed of an electromechanical CU connected to a hydraulic OC that adopts the same circumferential occlusion mechanism as the AMS 800. It's a crucial consideration when comparing artificial urinary sphincters. Indeed, the mechanism by which urethral compression is applied directly influences both the closure pressure generated and its distribution along the urethral wall. As demonstrated by Marziale et al., circumferential occlusion provides a distinct sealing behavior compared to clamping systems, particularly within the physiological pressure range of the lower urinary tract. These findings were supported by finite element modeling and validated through benchtop experimentation using urethral phantoms, allowing precise comparison between occlusion strategies [19]. The biomechanical behavior of the urethra under various occlusive loads had previously been characterized in detail through numerical modeling approaches, which served as the foundation for this comparative analysis [20].

### 4.4 UroActive Expected Benefits

As mentioned in the introduction, the occlusion pressure applied by the AMS800 cuff to the urethra is determined at implantation by the choice of BRP. Urethral occlusion pressure is therefore continuous and non‐modifiable after device activation.

UroActive is an electronic device which embeds automatic functions and can be controlled remotely by the patient via a remote control and configured by the physician via an external clinician programmer. Thus, device pressure could be changed during the day according to patient needs and could be adjusted by the physician during follow‐up visits according to patient continence satisfaction. Lowering the occlusive pressure during phases of low activity, patient sleep for example, could lead to urethral preservation. In a 2001 study conducted between the Mayo Clinic (Rochester, Minnesota, USA) and Baylor (Waco, Texas, USA), Elliott et al. show that nocturnal deactivation of the AMS800 could decrease the risk of urethral atrophy and urinary incontinence, although these results were not statistically significant [21].

The expected benefits of these features are to improve therapy effectiveness while decreasing device revision or explantation rate.

## 5 Conclusions

UroActive is a new electronic AUS which can be remotely controlled, so that the urethral occlusive pressure can be changed during the day and adjusted to the level of continence, even after device implantation and activation.

This pre‐clinical study demonstrates that the MUCPs achieved with UroActive are across a range that encompasses the values provided by the AMS800 PRBs for both male and female cadavers. Additionally, the strong positive correlation between set device pressures and MUCP observed with UroActive underscores its precise pressure control capabilities.

This study suggests that the UroActive AUS has the potential to offer a new adjustable option for managing stress urinary incontinence, with remote control capabilities that may enhance patient outcomes.

## Author Contributions

All authors participated, according to their personnal skills, in the protocol development, the study achievement, the data analysis and the writing of the manuscript. No AI was used.

## Conflicts of Interest

Pierre Mozer is co‐founder of Uromems. Aurelien Beaugerie and Emmanuel Chartier‐Kastler are consultants for Uromems.
